# The Cause and Prevention of Yellow Fever, Contained in the Report of the Sanitary Commission of New Orleans

**Published:** 1855-10

**Authors:** 


ART. VI.—*The Cause and Prevention of Yellow Fever, contained in the Report of the Sanitary Commission of New Orleans.* By E. H. BARTON, A. M., M. D., Chairman of the Sanitary Commission, President of the Louisiana State Medical Society, and of the New Orleans Academy of Sciences, etc. Philadelphia: Lindsay & Blakiston. 1855.

This is a reproduction, in a permanent and substantial form, of that memorable report hitherto favorably noticed in our pages, together with a suppliment in the form of a paper read before the N. O. Academy of Sciences, as a reply to the reviewer of the N. O. Medical Journal.

The reprinting of this report was almost a necessity, owing to the careless manner in which it was originally issued at New Orleans, as well as to its great importance, which demanded a larger circulation than that obtained by the original document, which was, we believe, only circulated gratuitously.

We must be content for the present to omit farther notice, hoping ere long to find space for a full consideration in connection with certain tables comparing the climatic conditions of the present summer, now in course of preparation. These tables will afford some data for the fair appreciation of Dr. Barton's theory, and will enable us to furnish an intelligent review.

In the meantime we wish that every one of our subscribers would purchase a copy of this report. We do not know that it is for sale here, but we will gladly act as agent for any one wishing to procure it from the publishers. Its price is within the limits of every ones ability.

